# Measuring the apparent diffusion coefficient in primary rectal tumors: is there a benefit in performing histogram analyses?

**DOI:** 10.1007/s00261-017-1062-2

**Published:** 2017-02-03

**Authors:** Miriam M. van Heeswijk, Doenja M. J. Lambregts, Monique Maas, Max J. Lahaye, Z. Ayas, Jos M. G. M. Slenter, Geerard L. Beets, Frans C. H. Bakers, Regina G. H. Beets-Tan

**Affiliations:** 1 Department of Radiology, The Netherlands Cancer Institute, P.O. Box 90203, 1006 BE Amsterdam, The Netherlands; 2 GROW School for Oncology and Developmental Biology- Maastricht University, Universiteitssingel 40, 6229 ER Maastricht, The Netherlands; 3 Department of Radiology, Maastricht University Medical Centre, P.O. Box 5800, 6202 AZ Maastricht, The Netherlands; 4 Department of Surgery, The Netherlands Cancer Institute, P.O. Box 90203, 1006 BE Amsterdam, The Netherlands

**Keywords:** Rectal cancer, Apparent diffusion coefficient, Histogram analysis, Prognostic marker

## Abstract

**Purpose:**

The apparent diffusion coefficient (ADC) is a potential prognostic imaging marker in rectal cancer. Typically, mean ADC values are used, derived from precise manual whole-volume tumor delineations by experts. The aim was first to explore whether non-precise circular delineation combined with histogram analysis can be a less cumbersome alternative to acquire similar ADC measurements and second to explore whether histogram analyses provide additional prognostic information.

**Methods:**

Thirty-seven patients who underwent a primary staging MRI including diffusion-weighted imaging (DWI; b0, 25, 50, 100, 500, 1000; 1.5 T) were included. Volumes-of-interest (VOIs) were drawn on b1000-DWI: (a) precise delineation, manually tracing tumor boundaries (2 expert readers), and (b) non-precise delineation, drawing circular VOIs with a wide margin around the tumor (2 non-experts). Mean ADC and histogram metrics (mean, min, max, median, SD, skewness, kurtosis, 5th–95th percentiles) were derived from the VOIs and delineation time was recorded. Measurements were compared between the two methods and correlated with prognostic outcome parameters.

**Results:**

Median delineation time reduced from 47–165 s (precise) to 21–43 s (non-precise). The 45th percentile of the non-precise delineation showed the best correlation with the mean ADC from the precise delineation as the reference standard (ICC 0.71–0.75). None of the mean ADC or histogram parameters showed significant prognostic value; only the total tumor volume (VOI) was significantly larger in patients with positive clinical N stage and mesorectal fascia involvement.

**Conclusion:**

When performing non-precise tumor delineation, histogram analysis (in specific 45th ADC percentile) may be used as an alternative to obtain similar ADC values as with precise whole tumor delineation. Histogram analyses are not beneficial to obtain additional prognostic information.

In recent years, diffusion-weighted imaging (DWI) has increasingly found its way to the clinical practice of magnetic resonance (MR) imaging in rectal cancer. Its value has particularly been demonstrated in the restaging setting to assess the response of the primary tumor to neoadjuvant chemoradiotherapy (CRT) and determine whether or not a residual tumor mass is still present within the post-radiation fibrosis [1–3]. In this setting, the addition of DWI has been shown to improve the sensitivity for tumor restaging after CRT by >30% in a recent meta-analysis [4].

Furthermore, in research settings, several studies have shown that quantifying the diffusion of rectal tumors by measuring the apparent diffusion coefficient (ADC) may be used as an imaging biomarker. This could be beneficial in clinics to predict prognostic factors such as nodal stage, mesorectal fascia (MRF) involvement, and histological differentiation grade [5–8]. Moreover, ADC may have value to predict therapeutic response [9–13], which (in the future) could impact treatment stratification. For example, neoadjuvant treatment may be further tailored depending on the anticipated treatment response. Moreover, accurate response evaluation can benefit the selection of good responding patient who can be candidates for minimally invasive follow-up treatments such as local excision or watchful waiting.

ADC values are most often expressed as the mean ADC, which can be acquired either from a single tumor slice [14, 15], from tumor sample measurements [3, 5], or by manually delineating the whole tumor volume on the diffusion-weighted images [1, 12, 16, 17]. The latter approach is most commonly advocated and has been shown to provide the most reproducible results [18, 19]. As such, it is now considered more or less the standard reference method of choice to acquire tumor ADC measurements. It is, however, a labor-intensive and time-consuming method, which is one of the factors that hamper the translation of the use of quantitative ADC measures from research settings to clinical practice.

Hypothetically, an alternative approach to obtain whole-volume ADC measurements could be to perform a non-precise tumor delineation, e.g., by roughly placing a circular volume of interest (VOI) with a wide margin around the tumor. This saves time, but non-tumoral tissues such as mesorectal fat, normal bowel wall and lumen, and the surrounding organs and muscle structures will be included in the VOIs, which will affect the ADC measurements. In theory, this effect may be compensated for by adding histogram analyses as a post-processing step. With histogram analysis, we analyze the spectrum of ADC values obtained from all voxels within the VOI. By doing so, we can not only extract the mean ADC values, but also, for example, calculate the minimum and maximum values and different percentile ranges. In principle, this information may be used to specifically focus on those ADC values within the VOI representing tumor (which will typically be the lower ADC values within the spectrum), for example by extracting only lower percentile values, thereby filtering out ‘noise’ from other included tissues. This could allow radiologist to save time with the delineation process whilst in principle obtaining the same ADC information. In addition, adding histogram analysis provides information about the distribution of ADC values within the tumor, which can potentially offer valuable additional insights into tumor structure and heterogeneity. This information could be of added benefit to predict response and prognosis, as has been suggested by previous authors [9, 10].

Therefore, the aim of this study was twofold. The primary aim was to test—on primary staging (pre-treatment) DWI-MRI—if histogram ADC analysis can be used to compensate non-precise tumor delineation and may be used as an alternative method to acquire similar ADC values as would normally be derived from precise whole-volume tumor delineation as the current ‘standard of reference.’ The secondary aim was to evaluate if histogram analysis provides valuable additional information to predict treatment outcome and prognosis.

## Materials and methods

### Patients

Forty-four patients diagnosed with and treated for rectal cancer at Maastricht University Medical Centre between October 2012 and June 2014 were considered for inclusion in this retrospective study. The study was approved by the local ethical institutional review board. Due to the retrospective nature of the study, informed consent was waived. Inclusion criteria were (a) biopsy-proven non-mucinous type rectal adenocarcinoma, (b) availability of a primary staging MRI including DWI (with a standardized acquisition protocol at 1.5 T), and (c) availability of follow-up data on treatment and outcome. Seven patients were excluded for the following reasons: severe artifacts on DWI, e.g., susceptibility artifacts due to air or metal prostheses (*n* = 5), multiple tumor sites in the rectum (*n* = 1), and prior pelvic radiation in (*n* = 1). This left a final study population of 37 patients.

### MR Imaging

All patients underwent a primary staging MRI at 1.5T MRI (Ingenia system, Philips Medical Systems, Best, The Netherlands), using a phased-array 16-channel body coil. The patients did not receive any bowel preparation. An intravenous bolus injection of 20 mg of butylscopolamine (Buscopan^®^, Boehringer Ingelheim bv, Ingelheim, Germany) was administered intravenously to reduce peristaltic movement. The standard imaging protocol included standard two-dimensional T2-weighted (T2 W) fast spin-echo sequences in 3 orthogonal directions (with the transverse images angled perpendicular and the coronal images angled parallel to the tumor axis as identified on the sagittal scan), and an axial echo planar imaging (EPI) DWI sequence angled in the same plane as the T2W transverse images. The DWI sequence was performed with spectral attenuated inversion recovery (SPAIR) fat suppression (*b* values 0, 25, 50, 100, 500, 1000 s/mm^2^; TR/TE 4147/66 ms; EPI factor 77; 5 number of signals acquired; 1.82 × 2.26 × 5.00 mm acquisition voxel size, 20 slices, slice gap of 0.5 mm; acquisition time of 6:44 min). Apparent diffusion coefficient maps were automatically generated by the operating system, using a mono-exponential decay model including all six *b* values.

### Precise and non-precise tumor delineation

**Fig. 1:**
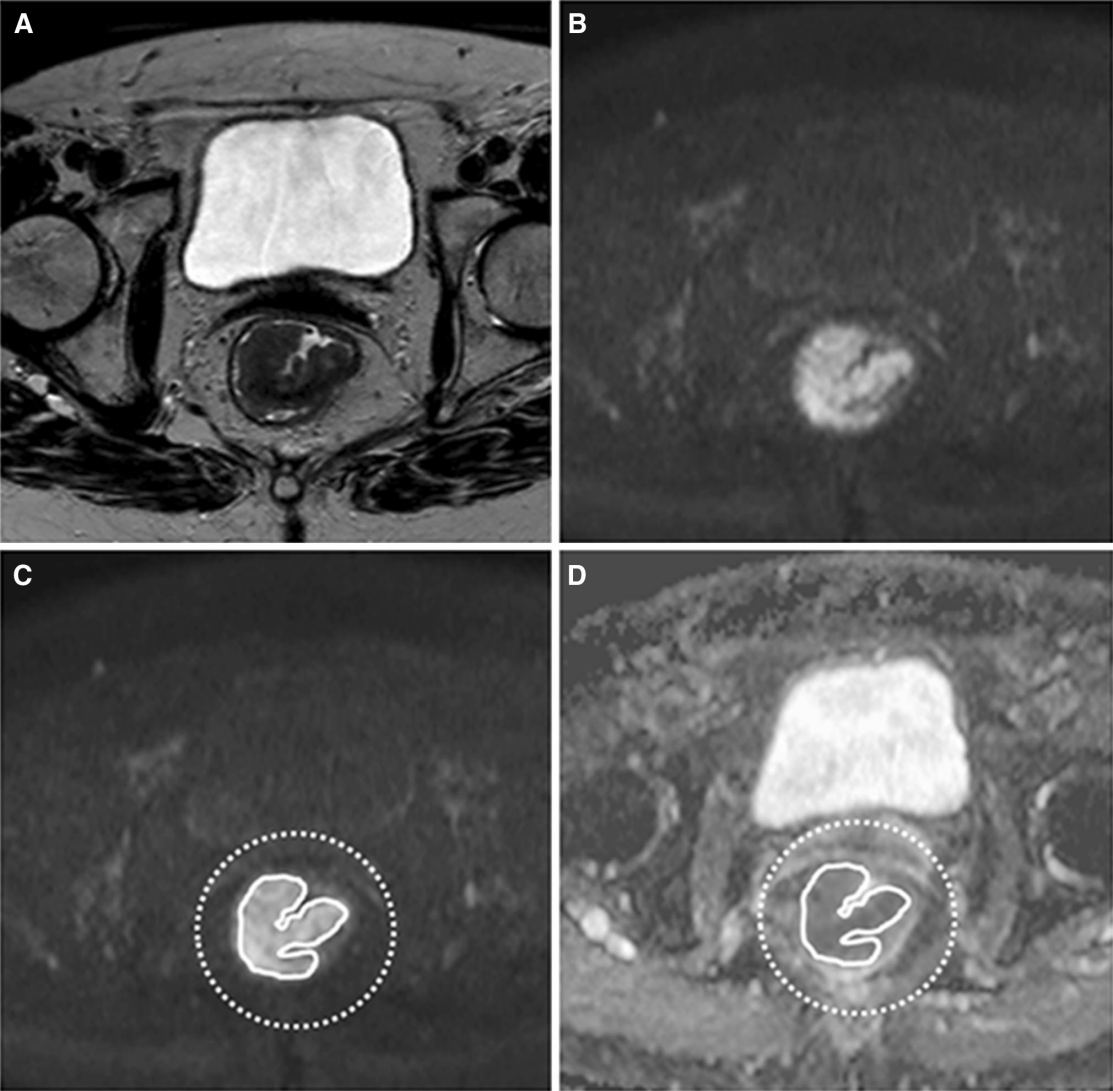
Example of the MR exam of an 82-year-old female patient. **A** Axial T2W image shows a semi-circular tumor, **B** the b-1000 DWI shows high signal in the tumor area, **C** example of the precise delineation (*solid line* performed by reader 1) and the non-precise delineation (*dotted line* performed by reader 3), the latter including both tumor and surrounding tissues, and **D** both delineations transferred to the ADC map

All primary staging MR images were transferred to an offline workstation for tumor delineation, which was performed using the freely available program MedView (Github clmedview, Maastricht, the Netherlands). Volumes-of-interest (VOIs) were drawn on the high-*b*-value (b1000) diffusion images in two ways: (a) precise delineation and (b) non-precise delineation. For the precise delineation, two experienced radiologists (R1 and R2; both with 8 years of experience in reading rectal MRI) manually closely traced the tumor boundaries on each consecutive slice to include the whole tumor volume. For the non-precise delineation, two additional non-expert resident-level readers (R3 and R4; both with no specific previous experience in reading rectal MRI) drew a circular/oval VOI with a margin around the tumor on each slice. The T2W images were at the disposal of all four readers for anatomical reference. An example illustrating the two delineation methods is shown in Fig. 1. The time required to perform the delineations was recorded in a representative sample of *n* = 18 study patients (for 1 expert and 1 non-expert reader) in order to quantify the potential reduction in measurement time.

### Histogram metrics

VOIs were transferred from the b1000 diffusion images to the ADC map to calculate mean ADCs and histogram metrics. Histogram plots were generated using a dedicated script written in MATLAB (The MathWorks Inc., Natick, MA, 2000) by one of the authors (JMGMS). The following histogram parameters were calculated: minimum, maximum, mean, median (50th percentile), standard deviation (SD), skewness, kurtosis, and every fifth percentile (5th–95th). The total volume (cm^3^) of each VOI was also recorded.

### Outcome parameters

Various prognostic and therapeutic outcome parameters were collected: (a) from the primary staging MRI reports, the cT stage, cN stage, and mesorectal fascia (MRF) involvement; (b) from the clinical patient database, the presence of distant metastases, neoadjuvant, and surgical procedures; (c) from the pathology reports (of the biopsy and surgical specimens), the tumor differentiation grade; and (d) in patients undergoing a long course of neoadjuvant treatment, the final treatment response defined as the tumor regression grade (TRG; method of Mandard) assessed at histopathology after surgery, where TRG 1–2 was considered a good response and TRG 3–5 a poor response [20].

### Statistical analysis

Statistical analyses were performed using the Statistical Package for the Social Sciences (SPSS, version 23.0, Inc., Chicago, IL). The intraclass correlation coefficient (ICC; using a two-way mixed method with absolute agreement for single measures) was used to calculate the agreement between the different readers and delineation methods. ICCs were also used to compare the various ADC histogram metrics of the non-precise delineation to the mean ADC of the precise delineation (being the most commonly used parameter in previous literature and therefore serving as the ‘standard of reference’). Agreement was additionally assessed using Bland–Altman statistics. Independent sample *T* tests (or Mann–Whitney *U*/Wilcoxon Rank test in case of non-normally distributed data) were used to compare the mean ADC values and various histogram metrics between (1) cN0 vs. cN+ patients, (2) cMRF− vs. cMRF+ patients, (3) patients with vs. without metastases, (4) tumors with good–moderate vs. poor differentiation grade at histopathology, and (5) good (TRG 1–2) vs. poor (TRG 3–5) responders. The Holm–Bonferroni correction method was applied to correct for multiple testing [21]. A Wilcoxon Rank test was performed to compare the delineation time between the two methods. *P* values ≤0.05 were considered statistically significant.

## Results

### Patient and treatment characteristics

Of the 37 study patients, 28 were male and nine female. Median age was 72 (range 29–86). Initial tumor stage on MRI was cT1–2 in 7 patients, cT3 in 27 patients, and cT4 in 3 patients. Eleven patients were cN0 and 26 were cN+. Eight patients had distant metastasis. Fifteen patients with non-locally advanced tumors underwent surgery without neoadjuvant treatment or immediately after a short course of 5 × 5 Gy. Seventeen locally advanced patients underwent a long course of CRT (28 × 1.8 Gy radiotherapy with 2 × 825 mg/m^2^/d capecitabine) or 5 × 5 Gy with a prolonged waiting interval before surgery. Five patients received palliative care.

### Precise vs. non-precise delineation method

**Fig. 2:**
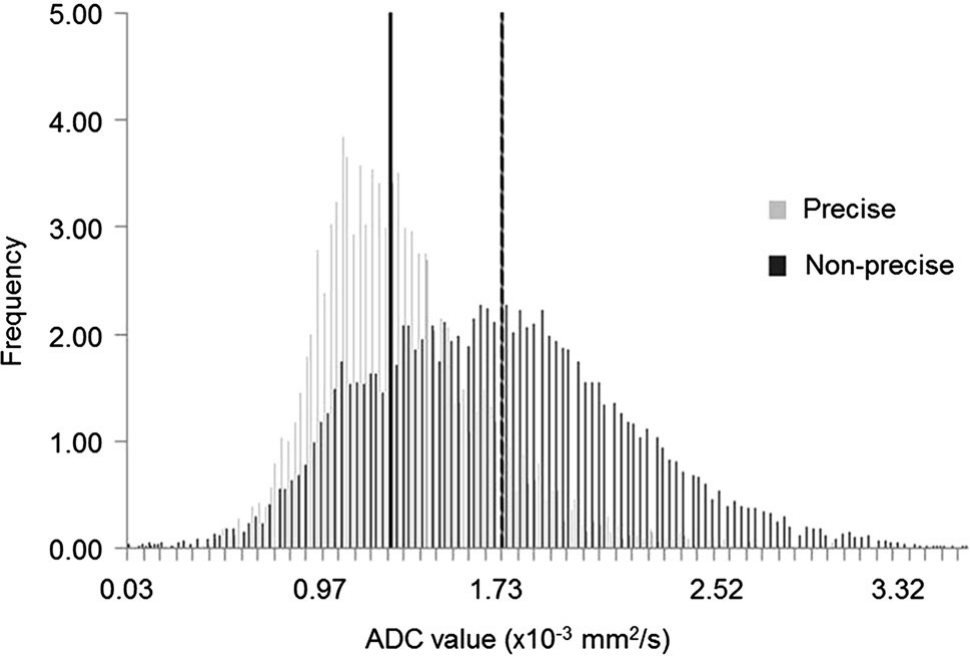
Example of the normalized histograms for the precise and non-precise delineation in the same patient. The *vertical lines* in *bold* represent the mean ADC per method (*solid line* indicating a mean ADC of 1.13 × 10^−3^ mm^2^/s for the precise delineation and the *dotted line* indicating a mean ADC of 1.64 × 10^−3^ mm^2^/s for the non-precise delineation). These normalized histograms show that the ADC values of the non-precise delineation are much more spread out due to the inclusion of other tissues, resulting in a higher mean ADC

VOIs, mean ADCs, and histogram metrics derived from the primary staging MRIs are provided in Table 1. The mean volume of the VOIs used to calculate the ADCs was 3.22 cm^3^ (R1) and 2.86 cm^3^ (R2) for the precise delineation method vs. 8.65 cm^3^ (R3) and 9.63 cm^3^ (R4) for the non-precise delineation method (*P* < 0.001). Mean ADC (×10^−3^ mm^2^/s) was 1.44 (R1) and 1.43 (R2) for the precise delineation vs. 1.51 (R3) and 1.51 (R4) for the non-precise delineation (*P* = 0.01–0.06). An example comparing the histograms of the precise and non-precise delineation methods is shown in Fig. 2. Table 2 shows the delineation times for the two measurement methods. Compared to precise delineation, non-precise delineation significantly reduced the delineation time for small, intermediate-sized as well as for the large tumors with a median reduction in measurement time of 28 up to 123 s per patient/tumor.

**Table 1:** Mean volumes, ADCs, and histogram metrics including interobserver agreement between the readers and between the delineation methods

	Precise delineation			Non-precise delineation			Precise vs. non-precise		
	Reader 1	Reader 2	ICC	Reader 3	Reader 4	ICC	R1 + R2^a^	R3 + R4^a^	ICC
Volume	3218	2858	0.91	8653	9629	0.88	3038	9141	0.49
Mean	1.44	1.43	0.98	1.51	1.51	0.96	1.43	1.51	0.64
Minimum	0.31	0.32	0.78	0.01	0.01	0.80	0.31	0.01	0.00
Maximum	3.18	3.18	0.82	3.58	3.61	0.95	3.18	3.60	0.64
SD	0.40	0.40	0.92	0.57	0.58	0.93	0.40	0.57	0.35
Skewness	0.66	0.67	0.87	0.15	0.17	0.83	0.67	0.16	0.12
Kurtosis	4.28	4.27	0.80	3.42	3.36	0.73	4.28	3.39	0.31
5th	0.89	0.88	0.93	0.58	0.57	0.90	0.88	0.57	0.20
10th	1.00	0.98	0.94	0.79	0.78	0.91	0.99	0.78	0.26
15th	1.07	1.05	0.96	0.94	0.92	0.90	1.06	0.93	0.35
20th	1.13	1.11	0.97	1.05	1.03	0.89	1.12	1.04	0.46
25th	1.18	1.16	0.98	1.14	1.12	0.90	1.17	1.13	0.57
30th	1.23	1.21	0.98	1.22	1.21	0.92	1.22	1.22	0.64
35th	1.28	1.25	0.98	1.30	1.29	0.93	1.27	1.29	0.67
40th	1.32	1.30	0.98	1.37	1.36	0.94	1.35	1.36	0.67
45%	1.37	1.34	0.98	1.44	1.43	0.94	1.36	1.43	0.67
50% (median)	1.40	1.38	0.98	1.50	1.50	0.95	1.39	1.50	0.65
55th	1.46	1.44	0.98	1.57	1.57	0.95	1.45	1.57	0.64
60th	1.51	1.49	0.98	1.64	1.64	0.96	1.50	1.64	0.63
65th	1.56	1.54	0.98	1.71	1.71	0.96	1.55	1.71	0.62
70th	1.62	1.60	0.98	1.79	1.79	0.96	1.61	1.79	0.62
75th	1.69	1.67	0.98	1.87	1.88	0.96	1.68	1.88	0.60
80th	1.76	1.75	0.98	1.97	1.98	0.97	1.75	1.98	0.58
85th	1.85	1.84	0.97	2.09	2.10	0.97	1.84	2.09	0.56
90th	1.96	1.96	0.96	2.24	2.25	0.97	1.96	2.25	0.53
95th	2.15	2.15	0.95	2.46	2.50	0.94	2.15	2.48	0.52

^a^Results are averaged between R1 + R2 and R3 + R4, respectively

**Table 2:** Delineation times for the non-precise and precise delineation methods

	Tumor volume (cm^3^)	Delineation time (s)			
		Precise	Non-precise	Difference	*P*
Small (*n* = 6)	0.57 (0.16–1.05)	47 (29–65)	21 (15–25)	28 (14–40)	0.03
Intermediate (*n* = 6)	2.28 (1.87–2.49)	101 (77–145)	31 (30–37)	71 (40–109)	0.03
Large (*n* = 6)	6.24 (4.99–18.32)	165 (107–292)	43 (34–64)	123 (68–228)	0.03

NB, numbers are medians with ranges provided in parentheses

Delineation time was measured for reader 1 (precise) and reader 3 (non-precise) in a representative sample of *n* = 18 tumors that were categorized into ‘small,’ ‘intermediate,’ and ‘large’ tumors by sorting all *n* = 37 tumors ascendingly according to their volume (derived from Table 1), dividing the group into 3 equal subsets based on tumor volume and randomly selecting a sample of *n* = 6 from each group

### Interobserver and intermethod agreement

**Fig. 3:**
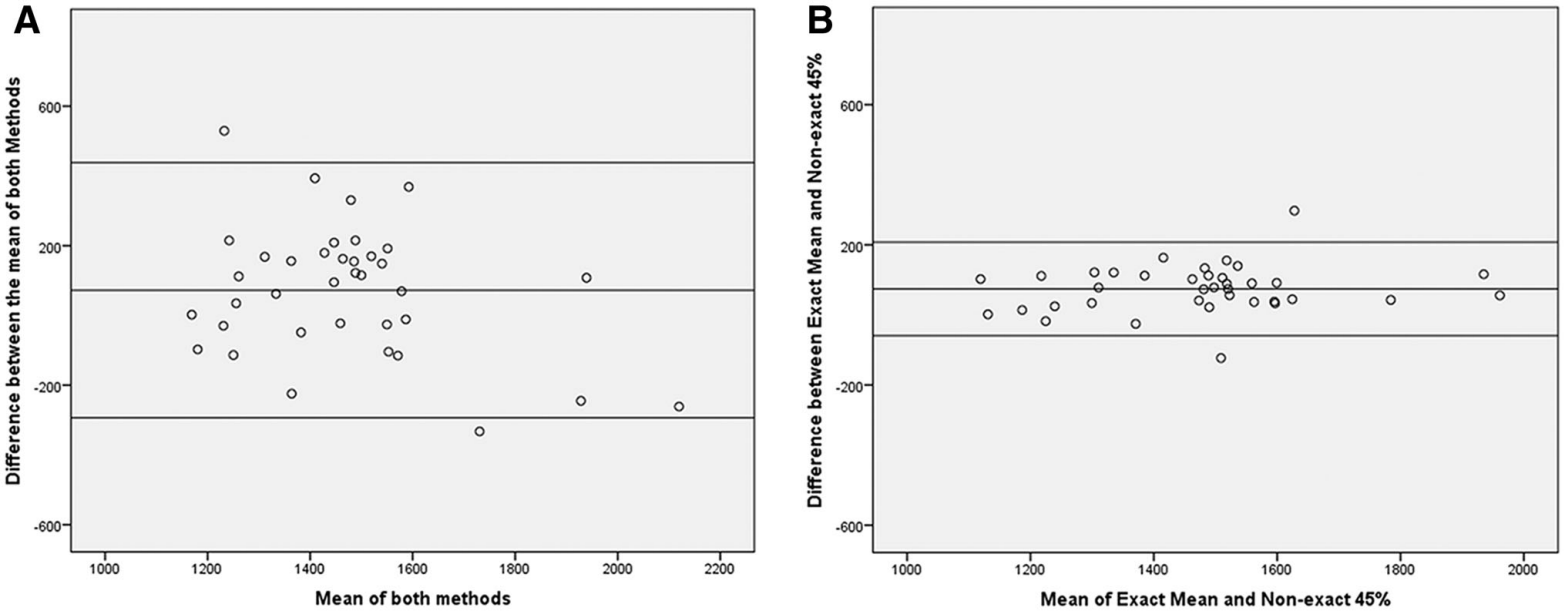
Bland–Altman plots showing the reproducibility for tumor ADC measurements plotted as the mean ADC of the two methods (*x*-axis) against the difference in ADC between the two methods (*y*-axis). The *middle line* represents the mean absolute difference (bias) between the two methods, while the *outer lines* represent the 95% confidence intervals (limits of agreement). Plot **A** compares the mean ADC derived from the precise delineation and non-precise delineation. Plot **B** compares the mean of the precise method and the 45th percentile of the non-precise method. Measurements were averaged for the two readers for both methods

ICCs between the different readers and delineation methods are given in Table 1. Interobserver agreement was excellent, both for the precise method (R1 vs. R2) and the non-precise method (R3 vs. R4) with ICCs ranging between 0.80 and 0.98. ICCs comparing the precise and non-precise methods were poor to good (ICC 0.00–0.67). Table 3 shows the correlations of the various histogram metrics of the non-precise method to the mean ADC of the precise method (as the standard of reference): best correlation was found for the 45th percentile ADC of the non-precise method (ICC of 0.71–0.75). Results for the mean ADC and 45th percentile measurements are illustrated using Bland–Altman plots in Fig. 3.

**Table 3:** ICCs constructed to explore which histogram parameter derived from non-precise delineation correlates best with the mean ADC of the precise delineation (as the standard of reference)

Precise (reference standard)	Mean ADC (R1):		Mean ADC (R2):	
Non-precise	R3	R4	R3	R4
Mean	0.63	0.63	0.63	0.62
Min	0.00	0.00	0.01	0.00
5th	0.02	0.01	0.02	0.01
10th	0.05	0.04	0.05	0.05
15th	0.09	0.09	0.09	0.09
20th	0.16	0.16	0.17	0.16
25th	0.27	0.26	0.28	0.26
30th	0.41	0.40	0.42	0.41
35th	0.55	0.55	0.56	0.55
40th	0.67	0.67	0.66	0.67
**45th**	**0.73**	**0.75**	**0.71**	**0.73**
50th (median)	0.72	0.74	0.71	0.72
55th	0.65	0.67	0.62	0.65
60th	0.55	0.58	0.53	0.55
65th	0.45	0.48	0.44	0.46
70th	0.36	0.38	0.36	0.37
75th	0.28	0.29	0.29	0.29
80th	0.22	0.22	0.22	0.23
85th	0.16	0.16	0.17	0.17
90th	0.11	0.11	0.12	0.12
95th	0.08	0.07	0.08	0.08
Max	0.02	0.02	0.02	0.02

NB, best results are printed in bold

### ADC histogram analysis vs. prognostic factors

In Table 4, VOIs, mean ADCs, and various histogram metrics are compared between different prognostic and response subgroups for both delineation methods. VOIs were significantly larger in the cN+ patients (both for the precise and non-precise delineation; *P* = 0.04) and in patients with MRF involvement on MRI (for the precise delineation; *P* = 0.04). Mean ADCs and the various histogram metrics were not significantly different between these subgroups. For the other outcome parameters (good vs. poor histopathological differentiation grade, good vs. poor response, metastasized vs. non-metastasized patients), none of the volume, ADC, or histogram metrics resulted in any significant differences between subgroups.

**Table 4:** Correlation of histogram metrics with prognostic and therapeutic outcome parameters for both methods

	cMRF involvement			cN stage			Differentiation grade^#^			Response after CRT^†^			Distant metastases		
	cMRF− *n* = 23	cMRF+ *n* = 14	*P*	cN− *n* = 11	cN+ *n* = 26	*P*	Good *N* = 19	Poor *N* = 3	*P*	Good *N* = 7	Poor *N* = 10	*P*	M− *N* = 29	M+ *N* = 8	*P*
Method 1 (precise)															
Volume	1995 ± 1686	4751 ± 5086	**0.04**	1388 ± 1195	3737 ± 4040	**0.04**	3903 ± 4761	1417 ± 1029	1	1698 ± 745	4080 ± 5151	1	2717 ± 3370	4202 ± 4360	1
Minimum	0.33 ± 0.30	0.28 ± 0.23	1	0.46 ± 0.32	0.25 ± 0.23	0.64	0.30 ± 0.27	0.49 ± 0.27	1	0.22 ± 0.23	0.30 ± 0.23	1	0.29 ± 0.26	0.40 ± 0.26	1
Maximum	2.96 ± 0.59	3.53 ± 0.46	0.05	2.83 ± 0.64	3.32 ± 0.54	0.20	3.17 ± 0.63	2.86 ± 0.74	1	2.88 ± 0.43	3.21 ± 0.55	1	3.13 ± 0.61	3.33 ± 0.59	1
Mean	1.37 ± 0.18	1.54 ± 0.32	1	1.50 ± 0.27	1.40 ± 0.25	0.96	1.45 ± 0.25	1.44 ± 0.18	1	1.50 ± 0.19	1.45 ± 0.26	1	1.44 ± 0.26	1.43 ± 0.23	1
Median	1.32 ± 0.19	1.50 ± 0.34	1	1.48 ± 0.27	1.35 ± 0.26	0.63	1.40 ± 0.26	1.41 ± 0.15	1	1.23 ± 0.19	1.41 ± 0.27	1	1.39 ± 0.28	1.38 ± 0.22	0.96
SD	0.36 ± 0.08	0.45 ± 0.12	0.22	0.35 ± 0.09	0.42 ± 0.10	0.22	0.39 ± 0.08	0.33 ± 0.05	1	0.38 ± 0.09	0.39 ± 0.08	1	0.39 ± 0.10	0.41 ± 0.10	1
Skewness	0.64 ± 0.46	0.71 ± 0.63	1	0.33 ± 0.38	0.81 ± 0.51	0.12	0.64 ± 0.39	0.34 ± 0.93	1	0.57 ± 0.48	0.62 ± 0.38	1	0.61 ± 0.49	0.88 ± 0.59	1
Kurtosis	4.08 ± 0.96	4.60 ± 1.92	1	3.59 ± 0.84	4.57 ± 1.50	0.45	3.90 ± 1.03	3.91 ± 1.09	1	4.03 ± 1.28	3.93 ± 0.90	0.89	4.11 ± 0.97	4.86 ± 2.42	1
5th	0.87 ± 0.16	0.90 ± 0.25	1	0.98 ± 0.18	0.84 ± 0.19	0.54	0.91 ± 0.16	0.93 ± 0.20	1	0.74 ± 0.30	0.91 ± 0.16	1	0.88 ± 0.16	0.88 ± 0.30	1
30th	1.18 ± 0.20	1.29 ± 0.29	1	1.31 ± 0.22	1.18 ± 0.24	0.56	1.22 ± 0.22	1.27 ± 0.18	0.98	1.14 ± 0.30	1.23 ± 0.23	1	1.23 ± 0.25	1.19 ± 0.22	1
45th	1.30 ± 0.21	1.45 ± 0.33	1	1.44 ± 0.25	1.32 ± 0.27	0.55	1.36 ± 0.25	1.37 ± 0.14	1	1.27 ± 0.29	1.37 ± 0.26	1	1.36 ± 0.28	1.33 ± 0.21	1
70th	1.54 ± 0.24	1.73 ± 0.40	1	1.67 ± 0.32	1.56 ± 0.32	0.46	1.32 ± 0.30	1.57 ± 0.19	1	1.51 ± 0.30	1.61 ± 0.31	1	1.62 ± 0.34	1.59 ± 0.26	1
95th	2.04 ± 0.30	2.32 ± 0.44	0.3	2.17 ± 0.38	2.17 ± 0.38	0.70	2.16 ± 0.37	2.02 ± 0.32	1	1.99 ± 0.31	2.15 ± 0.34	1	2.15 ± 0.40	2.15 ± 0.32	1
Method 2 (non-precise)															
Volume	7018 ± 3820	12,627 ± 10,419	0.36	5371 ± 3750	10,735 ± 8107	**0.04**	3275 ± 3327	11,109 ± 9973	1	7199 ± 3551	12,398 ± 11,173	1	8742 ± 7525	10,586 ± 7552	1
Minimum	0.01 ± 0.02	0.02 ± 0.02	0.48	0.01 ± 0.01	0.01 ± 0.02	1	0.02 ± 0.02	0.01 ± 0.02	1	0.01 ± 0.01	0.01 ± 0.02	1	0.01 ± 0.01	0.02 ± 0.02	1
Maximum	3.42 ± 0.55	3.89 ± 0.30	0.13	3.22 ± 0.57	3.75 ± 0.41	0.12	3.07 ± 0.54	3.61 ± 0.57	0.88	3.37 ± 0.31	3.55 ± 0.56	1	3.56 ± 0.51	3.72 ± 0.58	1
Mean	1.45 ± 0.18	1.59 ± 0.21	0.44	1.48 ± 0.19	1.52 ± 0.21	1	1.35 ± 0.20	1.52 ± 0.22	1	1.48 ± 0.09	1.54 ± 0.19	1	1.51 ± 0.20	1.52 ± 0.22	1
Median	1.45 ± 0.15	1.58 ± 0.23	0.50	1.50 ± 0.17	1.49 ± 0.21	1	1.39 ± 0.18	1.50 ± 0.20	1	1.47 ± 0.10	1.54 ± 0.18	1	1.50 ± 0.19	1.49 ± 0.22	1
SD	0.56 ± 0.12	0.59 ± 0.13	0.86	0.54 ± 0.11	0.59 ± 0.13	1	0.48 ± 0.02	0.58 ± 0.13	0.84	0.56 ± 0.10	0.55 ± 0.08	1	0.58 ± 0.13	0.53 ± 0.07	1
Skewness	0.04 ± 0.29	0.36 ± 0.53	0.54	0.14 ± 0.37	0.17 ± 0.44	1	0.19 ± 0.43	0.06 ± 0.28	1	0.07 ± 0.42	0.34 ± 0.49	1	0.13 ± 0.43	0.29 ± 0.36	1
Kurtosis	3.24 ± 0.61	3.63 ± 1.03	1	3.16 ± 0.46	3.48 ± 0.90	1	3.03 ± 0.55	3.28 ± 0.07	0.72	3.16 ± 0.52	3.08 ± 0.29	1	3.22 ± 0.65	3.96 ± 1.09	1
5th	0.52 ± 0.20	0.65 ± 0.27	0.54	0.52 ± 0.21	0.59 ± 0.24	1	0.49 ± 0.20	0.58 ± 0.27	1	0.54 ± 0.22	0.61 ± 0.23	1	0.54 ± 0.21	0.68 ± 0.29	1
30th	1.17 ± 0.16	1.28 ± 0.18	0.48	1.23 ± 0.17	1.21 ± 0.18	1	1.12 ± 0.22	1.22 ± 0.19	1	1.21 ± 0.17	1.26 ± 0.15	1	1.21 ± 0.17	1.24 ± 0.22	1
45th	1.39 ± 0.16	1.51 ± 0.22	0.42	1.44 ± 0.17	1.43 ± 0.20	1	1.33 ± 0.18	1.44 ± 0.20	1	1.42 ± 0.13	1.47 ± 0.17	1	1.43 ± 0.18	1.43 ± 0.22	1
70th	1.74 ± 0.19	1.87 ± 0.29	1	1.78 ± .21	1.80 ± 0.25	1	1.62 ± 0.17	1.80 ± 0.24	1	1.72 ± 0.07	1.83 ± 0.22	1	1.80 ± 0.24	1.75 ± 0.24	1
95th	2.41 ± 0.40	2.60 ± 0.32	0.55	2.34 ± 0.33	2.55 ± 0.39	0.88	2.08 ± 0.25	2.52 ± 0.42	0.65	2.28 ± 0.22	2.46 ± 0.31	1	2.50 ± 0.41	2.42 ± 0.27	1

NB, Values for each method are averaged for the two readers

^#^ ‘Good’ = good, good–moderate, and moderate differentiation, ‘Poor’ = poor or poor–moderate differentiation

^†^ ‘Good’ = tumor regression grade 1–2, ‘Poor’ = TRG 3–5

To correct for multiple testing, Holm–Bonferroni correction was performed (and mainly the most commonly reported percentile ranges were tested to reduce the number of testing variables). Significant results are printed in bold

## Discussion

The primary aim of this study was to assess the feasibility of calculating ADC values of rectal tumors at the time of primary staging using non-precise rectal tumor delineation combined with histogram analysis as an alternative to precise manual tumor delineation, aiming to simplify and speed up the delineation process. Precise volumetric delineation (typically performed by manual tracing of the tumor boundaries by expert readers) is the most commonly used method in current literature to calculate mean tumor ADCs and therefore in a way considered the current ‘standard of reference’ method. The benefit of a non-precise delineation (e.g., simply placing a circular ROI with a margin around the tumor area) is that it is faster and can be performed by non-experienced readers. The main drawback, however, is that tissues other than tumor such as the normal rectal wall, perirectal fat, and adjacent organs will be included in the delineation, which will affect the mean ADC. Our hypothesis was that this effect may be overcome by adding histogram analysis to filter out these effects and specifically focus on ADC values of the tumor within the histogram in order to acquire similar ADCs as would have normally been derived by calculating the mean ADC from a precise delineation.

Our results show that when using histogram post-processing in such a way, the 45th percentile ADC from the non-precise delineation showed the best correlation with the mean ADC from the precise delineation as the standard of reference (ICC 0.71–0.75). Results without the addition of histogram post-processing were considerably poorer with an ICC of only 0.64 between the mean ADCs of the precise and non-precise methods. The main reason for this poorer correlation is that the non-precise delineation resulted in remarkably higher overall ADC values, which can be explained by the fact that voxels with relatively high ADCs, for example, from the bladder, seminal vesicles, prostate, and normal rectal wall were often included in the VOIs (see Figs. 1 and 2).

Although the use of the 45th percentile instead of mean ADC from the non-precise delineation thus improved the results, an agreement with a maximum ICC of 0.75 was still suboptimal, especially when comparing it, for example, to the ICC of 0.98 between the two readers for the precise delineation method. A previous study explored the use of (semi-)automated tumor segmentation (using computer algorithms) as an alternative method to overcome the problem of time-consuming and labor-intensive manual tumor segmentation in rectal cancer. Similar to the current study, the results of manual delineation were used as the reference standard. Although the main outcome was the VOI itself (and not the ADC as in our study), high ICCs of 0.91–0.97 for semi-automated tumor segmentation were reported [22]. It would seem logical to assume that such an approach (given the excellent agreement when comparing the VOIs) would also result in a good agreement in ADC measurements if these were to be derived from these (semi-)automatically generated VOIs. It was reported that the median delineation time decreased from 180–296 s for precise manual delineation to 41–69 s for semi-automated segmentation, which entails a considerable decrease in time and input required from readers [22]. In our study, delineation time also significantly reduced with the non-precise method to a median measurement time ranging between 21 and 43 s per tumor/patient depending on the tumor volume. This would make it a similarly or even more effective solution with regard to time efficacy with the added benefit that the non-precise delineations can be performed by non-experienced readers and do not necessarily require expert input. The clinically relevant question is, however, if and how different delineation methods affect the utility of the acquired ADC measurements as a prognostic imaging biomarker.

Therefore, the second aim of this study was to explore the prognostic value of ADC measurements derived from the two delineation methods. In addition, we aimed to evaluate whether the addition of histogram analysis provides valuable extra information. Previous studies have shown that mean ADC at primary staging may differentiate prognostically unfavorable tumor subtypes (e.g., tumors with MRF involvement, clinical N+ stage, tumor deposits, and poor differentiation grade) [5, 6]. In our study, we could unfortunately not reproduce these findings. Mean ADC values were not useful to differentiate between cMRF− and cMRF+ tumors, cN− and cN+ tumors, well and poorly differentiated histological tumor subtypes, or between patients with/without metastasized disease. Moreover, the addition of histogram parameters did not lead to improved results. For the prediction of treatment response in the subgroup of patients undergoing chemoradiotherapy, neither mean ADC nor any of the histogram parameters showed significant results to differentiate between the poor and good responders using the tumor regression grade at histopathology as the outcome. Results in the literature regarding this issue have also been conflicting. Some groups reported significantly lower mean ADC values in patients who showed a good response to treatment, suggesting that pre-treatment ADC may have potential to predict response, which could be of potential clinical benefit to tailor (neoadjuvant) treatment strategies depending on the anticipated response [11–13, 23–25]. Conversely, other groups found—similar to the current study—no significant differences in pre-treatment ADC between responders and non-responders [25–29]. To date, only a few studies have investigated the potential benefit of adding histogram post-processing to predict rectal tumor response. Nougaret et al. reported that histogram metrics did not add to median ADC values for the assessment of rectal tumor response after CRT [19]. Choi et al. found some promising results after CRT, with significant differences between poor and good responders after CRT for several histogram parameters (minimum ADC, 10th, 25th, 50th, and 75th percentiles) [10]. This was confirmed in another study by Cho et al. who reported significantly different 10th and 25th percentile values between responders and non-responders with better diagnostic performance compared to mean ADC [9]. However, similar to the findings of our current study, Choi et al. find no benefit in any of the ADC or histogram metrics for pre-treatment prediction of response [10].

Interestingly, the VOI was the only parameter that resulted in significant differences between some of the favorable and unfavorable (cN+, cMRF+) subgroups. Previous studies also showed superior results for DWI tumor volumetry compared to ADC measurements, albeit these studies focused on the assessment of tumor response after chemoradiotherapy rather than for predicting prognostic factors at primary staging. Pre-treatment DWI tumor volumes in those previous reports did not show any significant correlations with the final treatment outcome [1, 30], which is in line with our current findings.

Our study had some limitations, the first of which being its retrospective nature and the relatively small number of patients. Second, for some of the study patients not all of the outcome variables were available (for example, because patients underwent palliative treatment and did not proceed to surgery). Moreover, the prognostic outcome factors (such as N stage and MRF involvement) were primarily based on the MRI staging result rather than histopathology. This method—also previously used by other authors [5, 6]—was chosen, since part of the study patients underwent neoadjuvant treatment before surgery and in these patients the final histopathology will no longer reflect the primary tumor stage. We, however, acknowledge that the clinical staging is a subjective measure that will be influenced by known limitations of MRI in assessing these factors, as well as radiologists’ experience. Third, mean ADC derived from a precise expert delineation was arbitrarily defined as a standard of reference, because it is the measure that is currently most widely used in published reports. We acknowledge, however, that this is a subjective standard of reference that will vary, for example, depending on the DWI image protocol (e.g., acquisition parameters, patient preparation, etc.) as well as the experience of the readers. Finally, we performed our analyses based on the assumption that the ADC values from both the precise and non-precise methods are normally distributed within patients (as illustrated in Fig. 2), which would make it acceptable to identify the non-precisely measured 45th percentile as the best surrogate measurement for the mean ADC from the precise method. This was, however, not tested for each individual patient.

In conclusion, the 45th percentile ADC of the histogram derived from non-precise delineation correlates well with the mean ADC of the precise method and may thus be used as an alternative measure. In our study, we could not confirm the previously reported potential value of ADC measurements to predict the prognostic tumor profile or response to treatment. Moreover, histogram ADC analysis did not appear to provide any additional prognostic information. Tumor volume was the only parameter found to correlate with prognostic features (N+ and MRF+ status).
